# clotFoam: An open-source framework to simulate blood clot formation under arterial flow

**DOI:** 10.1016/j.softx.2023.101483

**Published:** 2023-08-03

**Authors:** David Montgomery, Federico Municchi, Karin Leiderman

**Affiliations:** a Department of Applied Mathematics and Statistics, Colorado School of Mines, 1500 Illinois St, Golden, CO 80401, United States of America; b Department of Mechanical Engineering, Colorado School of Mines, 1500 Illinois St, Golden, CO 80401, United States of America; c Department of Mathematics, University of North Carolina at Chapel Hill, 216 Lenoir Dr, Chapel Hill, NC 27599, United States of America; d Computational Medicine Program, University of North Carolina at Chapel Hill, 216 Lenoir Dr, Chapel Hill, NC 27599, United States of America

**Keywords:** Blood clotting, Platelet aggregation, Coagulation, Hemostasis, Multiscale modeling, OpenFOAM

## Abstract

Blood clotting involves the coupled processes of platelet aggregation and coagulation. Simulating clotting under flow in complex geometries is challenging due to multiple temporal and spatial scales and high computational cost. *clotFoam* is an open-source software developed in OpenFOAM that employs a continuum model of platelet advection, diffusion, and aggregation in a dynamic fluid environment and a simplified coagulation model with proteins that advect, diffuse, and react within the fluid and with wall-bound species through reactive boundary conditions. Our framework provides the foundation on which one can build more complex models and perform reliable simulations in almost any computational domain.

**Table:** Code metadata

Current code version	1.0
Permanent link to code/repository used for this code version	https://github.com/ElsevierSoftwareX/SOFTX-D-23-00244
Code Ocean compute capsule	
Legal Code License	GNU GPL V3
Code versioning system used	git
Software code languages, tools, and services used	C++, C, MPI, GNU Make
Compilation requirements, operating environments & dependencies	OpenFOAM-v9
If available Link to developer documentation/manual	
Support email for questions	dmontgomery@mines.edu

## 1. Motivation and significance

Blood clotting is the body’s response to prevent bleeding from an injured blood vessel. The clotting process involves two main components: platelet aggregation and coagulation. Platelet aggregation is a primarily physical process where platelets adhere to the injured vessel wall, and become activated by receptors that interact with proteins embedded in the wall. Activated platelets release agonists, such as adenosine diphosphate (ADP), which can activate and recruit more platelets to the injury where they begin to form a platelet plug. Coagulation is a biochemical process involving dozens of enzymatic reactions that occur in the fluid, on activated platelet surfaces, and on injured portions of the vessel wall. Coagulation features the interplay of positive and negative feedback loops that work collectively to encourage thrombus growth in a self-regulating manner. The reactions culminate in the generation of the enzyme thrombin on activated platelet surfaces, which is a strong platelet activation agonist, a key player in positive feedback, and converts fibrinogen into fibrin, which polymerizes and forms a stabilizing fibrin gel on the platelet plug. Platelets play a critical role in coagulation, as thrombin generation and inhibition is strongly regulated by their activated surfaces [1–4]. Thrombin generation is often used as a clinical indicator of healthy clotting, as without thrombin, a clot is typically leaky and unstable. The interested reader can find more information about the blood clotting process and previously developed mathematical models in a number of reviews published elsewhere [5–9].

Spatio-temporal continuum models that employ computational fluid dynamics (CFD) have gained widespread usage to study blood clotting in devices [10], in common microfluidic assays [11,12], in aneurysms [13] and bleeding [14,15], and to understand the effects of flow and transport on the clotting process overall [3,12,16,17]. A significant proportion of spatial-temporal models of clotting have been implemented using inhouse codes [3,15,16], commercial tools with high licensing costs [11], or are not publicly available [10,12,13,18], posing a challenge for researchers with limited resources. There is growing interest in open source and freely available software tools such as OpenFOAM [19]. This platform provides a versatile object-oriented toolkit for developing and constructing CFD software, featuring a diverse range of solvers and discretization schemes for general grids and parallel computing.

A few in-house codes of clotting models are freely available [20,21], but are quite model-specific and not necessarily made for other researchers to easily build upon. In this work we present clotFoam, a cell-centered finite volume solver that provides a flexible framework for simulating and easily extending a reduced model of blood clotting under flow. The software was developed with OpenFOAM libraries and can be perceived as an extension of the transient fluid solver icoFoam, which utilizes the PISO algorithm [22] for decoupling pressure from the fluid velocity. clotFoam incorporates several additions, including a Darcy term in the fluid equations, five advection-diffusion-reaction (ADR) equations that describe platelet aggregation and the release of ADP from platelet stores upon their activation, as well as twelve ADR equations that represent a reduced model of platelet surface-mediated coagulation. The coupling of reactions among the platelet and biochemical species is managed via object-oriented programming and a modified Runge-Kutta method, employing both field and patch field operations for the computation of reactions within the fluid and on the surface of the vessel wall. The following sections of this manuscript will detail the mathematical model employed by clotFoam, and provide a comprehensive description of the software. Two examples of the application of clotFoam in 2-D thrombosis and 3-D hemostasis simulations are presented. Finally, we discuss the potential implications and benefits of the clotFoam solver for the broader modeling and simulation community.

## 2. Model description

The *clotFoam* software is based on our previous model that used a continuum description of fluid, platelets, and platelet aggregation under flow [3]. The model describes blood as an incompressible Newtonian fluid that is governed by the Navier-Stokes-Brinkman equations:

(1) $$\begin{array}{rr} \rho \frac{\partial \vec{u}}{\partial t}+\rho (\vec{u}\cdot \nabla )\vec{u} & \;=-\nabla p+\mu \nabla^{2}\vec{u}-\mu \alpha \left(\theta^{B}\right)\vec{u}, \end{array}$$

(2) $$\begin{array}{rr} \nabla \cdot \vec{u} & \;=0, \end{array}$$

where $\vec{u}(\vec{x},t)$ is the fluid velocity, $p(\vec{x},t)$ is pressure, ρ is the fluid density, and μ is the dynamic viscosity. The Darcy term, $-\alpha \left(\theta^{B}\right)\vec{u}$, represents a frictional resistance to the fluid caused by a growing mass of bound platelets. The variable $\theta^{B}$ is the ratio of the sum of bound platelets to the maximum packing limit $P_{max}$. The permeability of the mass of bound platelets, $\alpha \left(\theta^{B}\right)$, decreases as $\theta^{B}$ increases, as it satisfies the Carman-Kozeny relation $\alpha \left(\theta^{B}\right)=$ $C_{CK}{\left(0.6\theta^{B}\right)}^{2}/{\left(1-0.6\theta^{B}\right)}^{3}$, where $C_{CK}=10^{6}{\;mm}^{-2}$.

Platelets are modeled as number densities (number per volume), eliminating the need for tracking individual platelets throughout the simulation. The dynamics of platelet aggregation are described generally by the following hindered ADR equation:

(3) $$\frac{\partial P^{k}}{\partial t}=-\nabla \cdot \left\{W\left(\theta^{T}\right)\left(\vec{u}P^{k}-D_{P}\nabla P^{k}\right)\right\}+S_{k}$$

where $P^{k}(\vec{x},t)$ is the k th platelet species and $S_{k}$ is a source/sink term that accounts for the transitions between different states of the platelet species. The full system of platelet aggregation equations are listed in Appendix A. To ensure that the number of platelets at a location $\vec{x}$ does not exceed a maximum packing limit, $P_{max}$, the platelet size is considered using three phenomenological functions: the hindered transport function $W\left(\theta^{T}\right)$, the adhesion region $H_{adh}(\vec{x})$, and the binding affinity function g(η). A detailed description of these functions can also be found in Appendix A.

The rates of activation of mobile-unactivated platelets by chemical agonists ADP and thrombin $\left(E_{2}\right)$ are assumed to satisfy Hill functions of the form $A(c)=k_{c}^{\text{pla }}\frac{c}{c^{*}+c}$. ADP is secreted by newly activated platelets over a period of 1–5 s after activation. The molar concentration of ADP satisfies:

(4) $$\frac{\partial [ADP]}{\partial t}=-\nabla \cdot \left\{\vec{u}[ADP]-D_{ADP}\nabla [ADP]\right\}+\sigma_{\text{release }},$$

where $D_{ADP}$ is the diffusion coefficient, and the source term is defined as:

(5) $$\sigma_{\text{release }}(\vec{x},t)=\int_{0}^{\infty }\;\overset{ˆ}{A}R(\tau )\frac{\partial }{\partial t}\left(P^{b,a}+P^{se,a}\right)(\vec{x},t-\tau )d\tau .$$

$\overset{ˆ}{A}$ is the total concentration of ADP released by an activated platelet, and $\overset{ˆ}{A}R(\tau )$ is the rate of release of ADP τ seconds after activation. The rate function, R(τ), utilized by *clotFoam* is similar to the one in our previous work [3], but here uses a bell curve centered at $3\;s,R(\tau )=\frac{1}{\sqrt{\pi }}exp\left(-(\tau -3{)}^{2}\right)$, and is normalized such that $\int_{0}^{\infty }\;R(\tau )d\tau =1$.

**Fig. 1.**
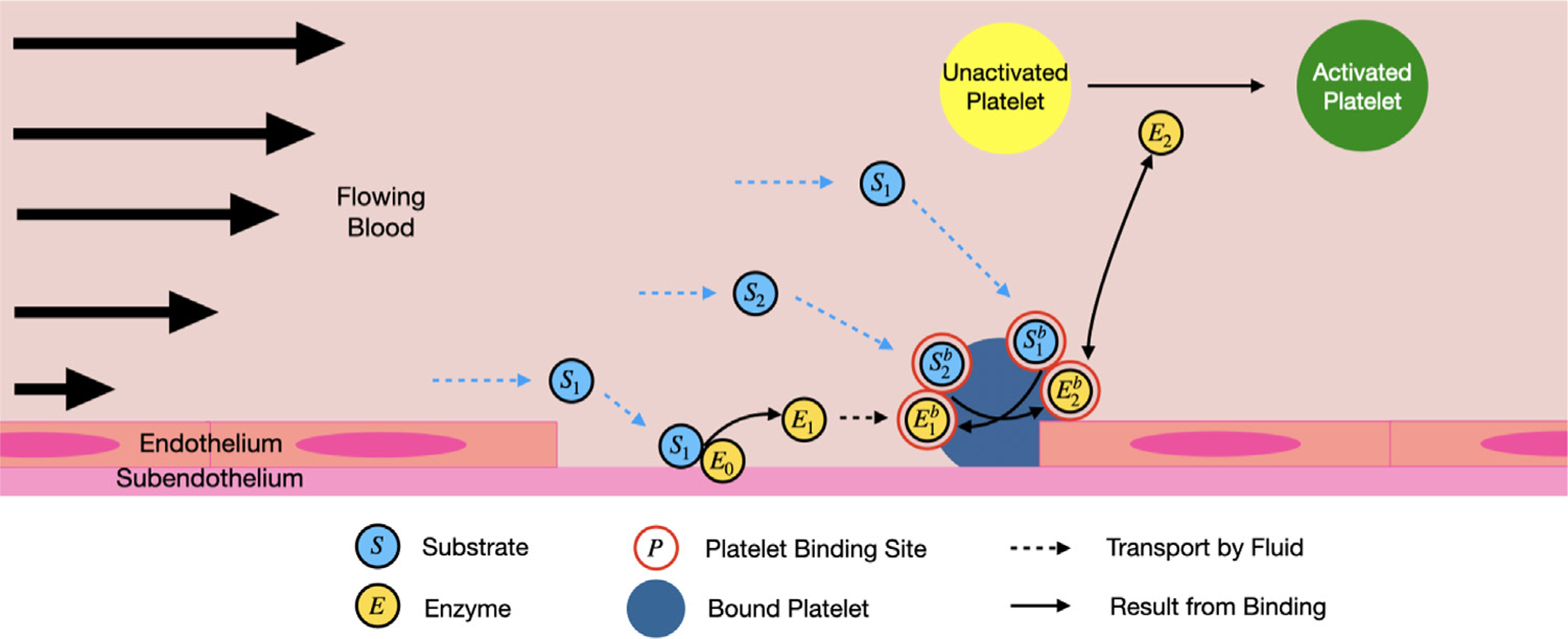
Schematic representation of the reduced model of thrombin generation with positive feedback. The light blue circles represent a substrate, yellow circles denote enzymes, and the red circles represent binding sites on the platelet surface. Dotted lines indicate transport by the fluid, while the solid lines represent binding interactions. *Note: Platelets have diameters near 3 μm, while endothelial cells are typically 50–70 μm in length and 0.1–10 μm in thickness* [23].

The reduced coagulation model consists of various biochemical species in molar concentrations and categorized as fluidphase, platelet-bound, or subendothelium-bound. In an effort to enhance the adaptability of clotFoam, the number of biochemical species has been reduced from 50 [3,16] to 12. This model is an extension of a previously published ODE model [1] that includes positive feedback and enzyme inhibition, two features necessary to capture the bursting thrombin behavior observed in coagulation. We extended the ODE model to a PDE model where reactions occur on two surfaces (subendothelium and activated platelets) instead of one, and the species are subjected to flow. The model is detailed in our Appendix B, and a schematic is presented in Fig. 1. The reduced model is summarized as follows where S denotes a substrate or zymogen, and E denotes an enzyme:

1. Fluid-phase substrate, $S_{1}$, comes into contact with enzyme, $E_{0}$, bound to the subendothelium. $E_{0}$ converts the substrate to an enzyme, $E_{1}$.
2. $E_{1}$ binds to the activated platelet surface and becomes the bound species, $E_{1}^{b}$.
3. Additional fluid-phase substrates, $S_{2}$, bind to the platelet surface and become $S_{2}^{b}$. Upon activation by $E_{1}^{b}$, the plateletbound substrates, $S_{2}^{b}$, are converted into a second plateletbound enzyme, $E_{2}^{b}$, which we consider to be similar to thrombin.
4. $E_{2}^{b}$ activates platelet-bound substrates, forming more enzymes in a positive feedback loop.
5. $E_{2}$ activates mobile unactivated platelets.

## 3. Software description

**Fig. 2.**
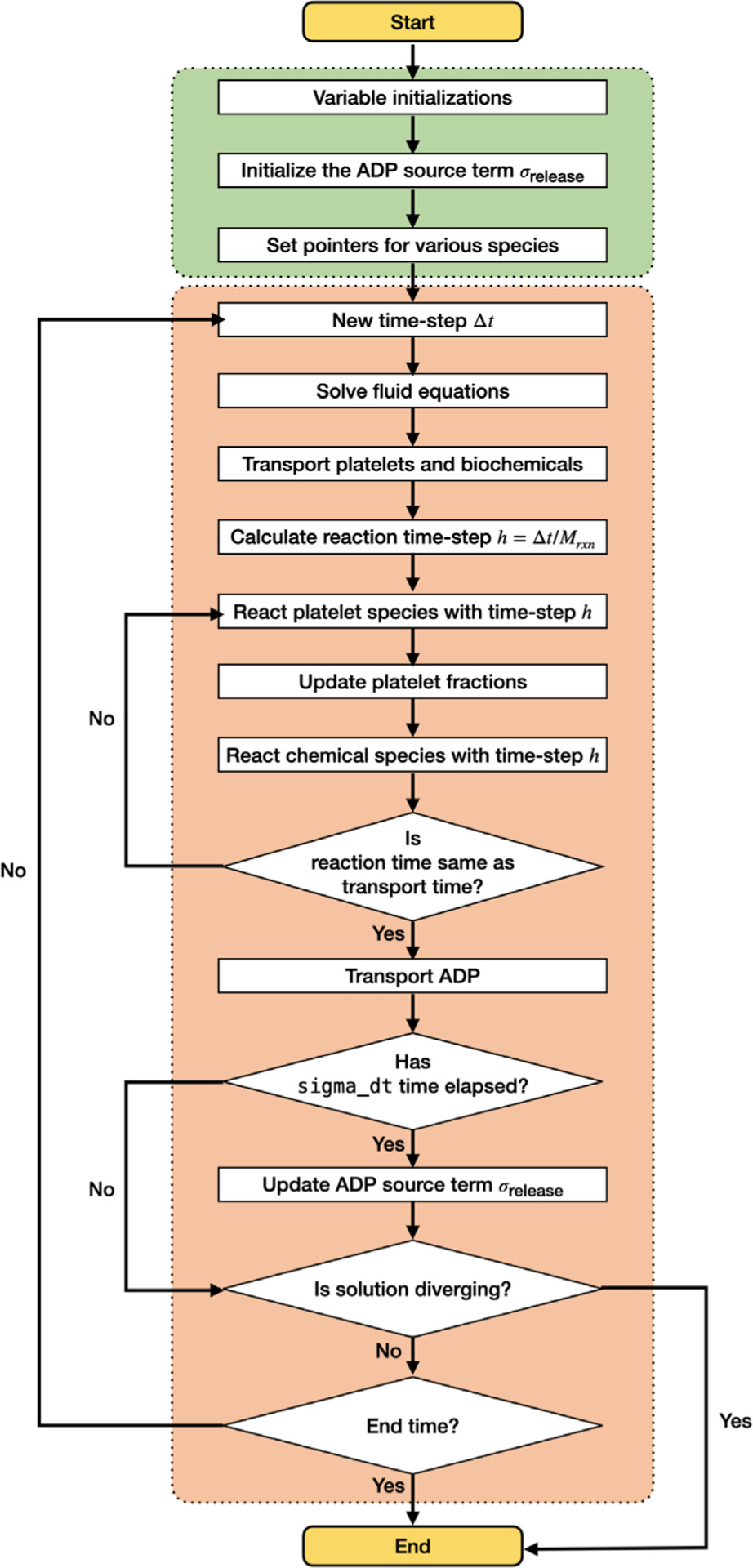
Flow chart of platelet-mediated coagulation solver within OpenFOAM.

*clotFoam* is an open-source software distributed under the GNU General Public License, compiled using the OpenFOAM-v9 libraries. The software can be compiled on any system where the OpenFOAM-v9 libraries are installed, and full installation instructions are provided in the repository. The code is written in C++ and can be adapted to simulate clotting in a wide variety of domains with few limitations to the mesh. The use of objectoriented programming enables the management of platelets and biochemical species as objects, which simplifies the implementation of more complex coagulation and clotting models. The repository contains two illustrative examples, with the expectation that its range will expand through community contributions and author updates as the framework develops further. clotFoam is fully parallelizable for high performance computing (HPC) using the message passing interface (MPI) framework. The solution algorithm employed by clotFoam is illustrated in Fig. 2 and is described in more detail in the following subsections.

### 3.1. Mesh requirements

**Fig. 3.**
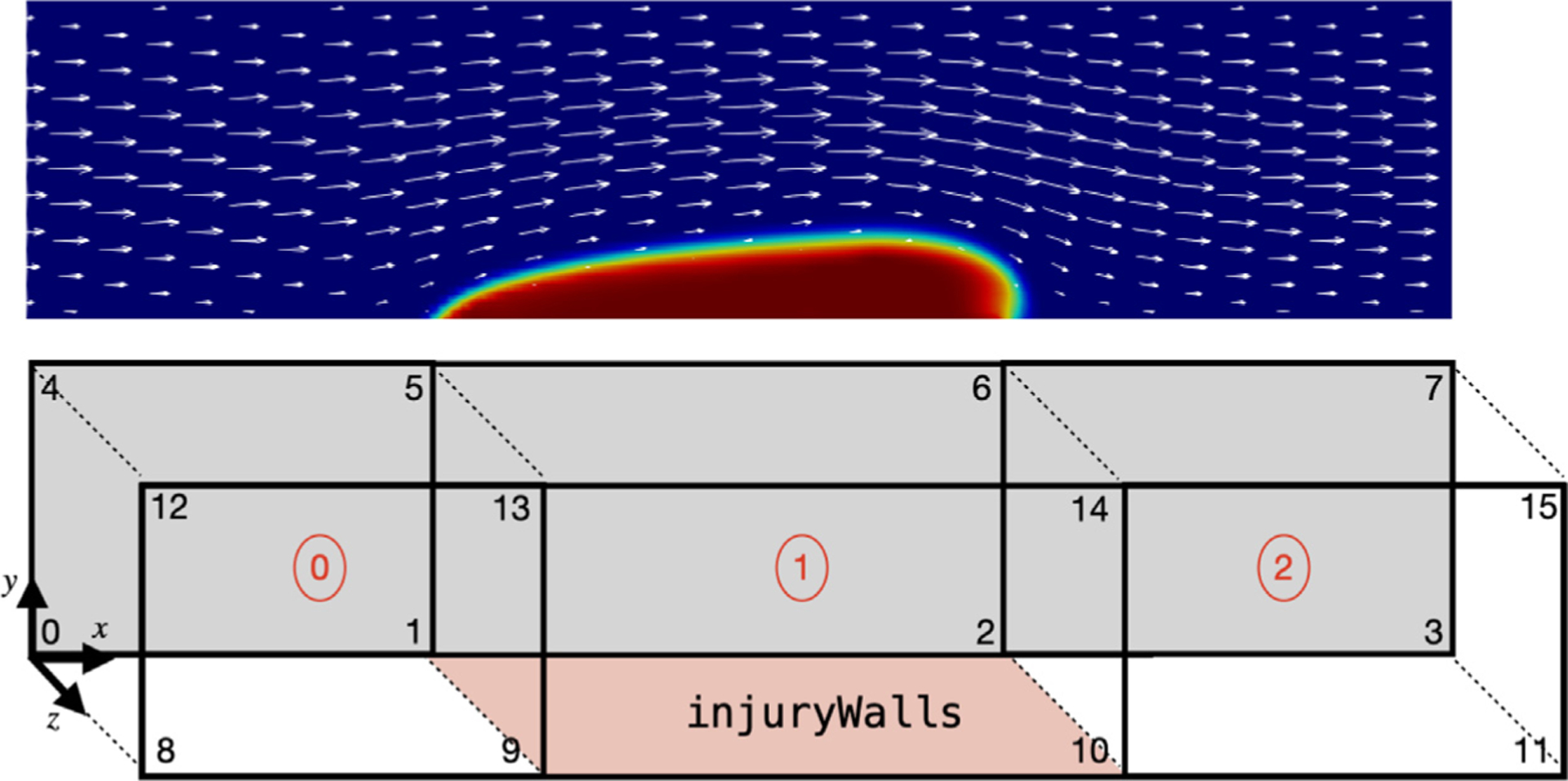
Example of thrombus growth in a corresponding domain defined with blockMesh using three blocks and 16 vertices. The injuryWalls patch is defined by vertices {1, 9, 10, 2}, which is used by *clotFoam* to determine the location of the reactive boundary conditions in the coagulation model.

To properly define the injury region, the mesh needs to satisfy two requirements. First, the reactive boundary conditions and subendothelium-bound species are defined exclusively on a patch known as “injuryWalls”. This patch is constructed to be the wall of an injury block within the domain using the blockMesh tool, as depicted in Fig. 3. Alternatively, users can use the topoSet tool to define the injuryWalls patch. Second, the mesh at the injury site must feature cell widths, heights, and depths no greater than a platelet diameter $P_{\text{diam }}$, which has a default value of 3μm. This requirement is a consequence of how the adhesion region $H_{\text{adh }}(\vec{x})$ is defined.

### 3.2. Numerical methods

The fluids solver in *clotFoam* is built upon OpenFOAM’s transient fluid solver icoFoam, which implements the PISO algorithm as a predictor and corrector method. In each time step, the momentum equation is solved once, followed by multiple pressure and velocity corrections. During the discretization of the Navier-Stokes-Brinkman Eqs. (1), the Darcy term is treated implicitly as a source term. This treatment involves utilizing the bound platelet fraction, $\theta^{B}$, from the previous time step. By incorporating the bound platelet fraction in this manner, we not only enhance the stability of the fluids solver but also ensure that the pressure corrections in the PISO algorithm are influenced by the presence of the porous media. Consequently, we are able to accurately capture the influence of the porous media on the fluid flow, resulting in simulations that exhibit improved reliability and robustness. Further details regarding other discretization schemes employed in this work can be found in Appendix D.

The model accounts for the transport of both platelet and biochemical species with equations that incorporate advection, diffusion, and reactions (ADR) with other species. The reactions occur on a smaller time scale than the transport processes, specifically, the software’s default reaction time-step is half of the transport time-step. As such, the software employs a fractionalstep method to decouple the transport from the reaction terms in the equations. Thus, the reaction equations are solved multiple times during each fluid time-step. The general ADR equation for each species is:

(6) $$\frac{\partial C_{i}}{\partial t}=-\nabla \cdot \left(\vec{v}C_{i}-D_{c}\nabla C_{i}\right)+R_{i}\left(C_{1},C_{2},\ldots ,C_{n}\right),$$

where $C_{i}$ is the i th species with $i=1,\ldots ,n,\vec{v}$ is a fluid velocity (not necessarily $\vec{u}$ from (1)), $D_{c}$ is the diffusion coefficient, and $R_{i}$ is a reaction term that can depend on multiple species.

A two-step fractional-step method is used to march Eq. (6) forward in time for each time step:

1. Solve the transport equation with a temporal step size Δt:
(7) $$\frac{\partial C_{i}}{\partial t}=-\nabla \cdot \left(\vec{v}C_{i}-D_{c}\nabla C_{i}\right).$$
2. Update the solution by solving the coupled reaction equations M_rxn times with a temporal step size $h=\Delta t/M_{rxn}$:
(8) $$\frac{\partial C_{i}}{\partial t}=R_{i}\left(C_{1},C_{2},\ldots ,C_{n}\right).$$

The transport equations are discretized using the finite volume method (FVM) as discussed in Appendix D, while the reaction equations are solved using a coupled fourth-order Runge-Kutta (RK4) method. The parameter M_rxn determines how many times the reaction equations are solved per fluid time-step, and is specified in the $FOAM_CASE/constant/inputParameters file. The default value is M_rxn = 2, however, it should be noted that this parameter is dependent on the specific problem and may need to be adjusted for flows with higher wall-shear rates.

In the mobile platelet equations described in Appendix A, the flux vector $\vec{j}=\vec{u}P-D_{P}\nabla P$ is scaled by a hindered transport function $W\left(\theta^{T}\right)$ to limit the transport of platelets near the growing thrombus. Prior to FVM discretization of the advective and diffusive fluxes, the total platelet fraction $\theta^{T}$ must be interpolated to the cell faces. The choice of interpolation method is determined by the mechanism of transport. By implementing a combination of interpolation schemes, the flux of platelets into a spatial location is effectively constrained, ensuring that the maximum value of the sum of all platelet species at a spatial location remains below or equal to a maximum packing density $P_{max}$. To interpolate the total platelet fraction for the advective flux, $W\left(\theta^{T}\right)\vec{u}P$, a downwind scheme is used with respect to the fluid velocity $\vec{u}$. This is because the fluid velocity is assumed to only be hindered by a thrombus that is downstream. Conversely, for the diffusive flux, $W\left(\theta^{T}\right)D_{P}\nabla P$, the total platelet fraction is interpolated using a localMax scheme, as the diffusion rate within the thrombus is expected to be smaller than the rate outside of the thrombus.

The ADP Eq. (4) is not solved using the fractional-step method, because the source term is updated infrequently. When platelets become bound, they release ADP into the fluid for up to 6 s. The secretion of ADP is modeled by the source term, $\sigma_{\text{release }}$, as defined in Eq. (5), and can be restricted to the interval τ∈[0,6] due to the bell-shaped distribution of R(τ). However, the computation of $\sigma_{\text{release }}$ is memory intensive as the number of newly bound platelets $\frac{\partial }{\partial t}\left(P^{b,a}+P^{se,a}\right)\left(\vec{x},t_{n}-\tau \right)$ must be stored in memory for up to 6 s. To reduce computational cost, a coarse discretization of τ is employed to calculate $\sigma_{\text{release }}$. The number of newly bound platelets are computed and stored at a user-specified interval Δτ. The discretization of $\sigma_{\text{release }}$ at time $t=t_{n}$ is then implemented using the trapezoid rule with a substitution $\tau =t_{n}-t^{\prime }$:

(9) $$\sigma_{\text{release }}\left(\vec{x},t_{n}\right)=\;\int_{0}^{\tau_{f}}\;\overset{ˆ}{A}R\left(t^{\prime }\right)\frac{\partial }{\partial t}\left(P^{b,a}+P^{se,a}\right)\left(\vec{x},t_{n}-t^{\prime }\right)dt^{\prime },\;=\;\;\int_{t_{n}-\tau_{f}}^{t_{n}}\overset{ˆ}{A}R\left(t_{n}-\tau \right)\frac{\partial }{\partial t}\left(P^{b,a}+P^{se,a}\right)(\vec{x},\tau )d\tau ,\;\approx \;\;\sum_{k=0}^{N_{\tau }-1}\overset{ˆ}{A}\frac{\Delta \tau }{2}\left\{R\left(t_{n}-\tau_{k}\right)\frac{\partial }{\partial t}\left(P^{b,a}+P^{se,a}\right)\left(\vec{x},\tau_{k}\right)\right.\;\left.+R\left(t_{n}-\tau_{k+1}\right)\frac{\partial }{\partial t}\left(P^{b,a}+P^{se,a}\right)\left(\vec{x},\tau_{k+1}\right)\right\},$$

where $\tau_{f}$ and Δτ are defined as sigma_Tf and sigma_dt respectively in the inputParameters dictionary. Lastly, the number of newly bound platelets is approximated as:

(10) $$\frac{\partial }{\partial t}\left(P^{b,a}+P^{se,a}\right)\left(\vec{x},t_{n}\right)\approx P_{max}\frac{\theta_{n}^{B}-\theta_{n-1}^{B}}{t_{n}-t_{n-1}}.$$

### 3.3. Managing platelet and biochemical species with polymorphism

**Fig. 4.**
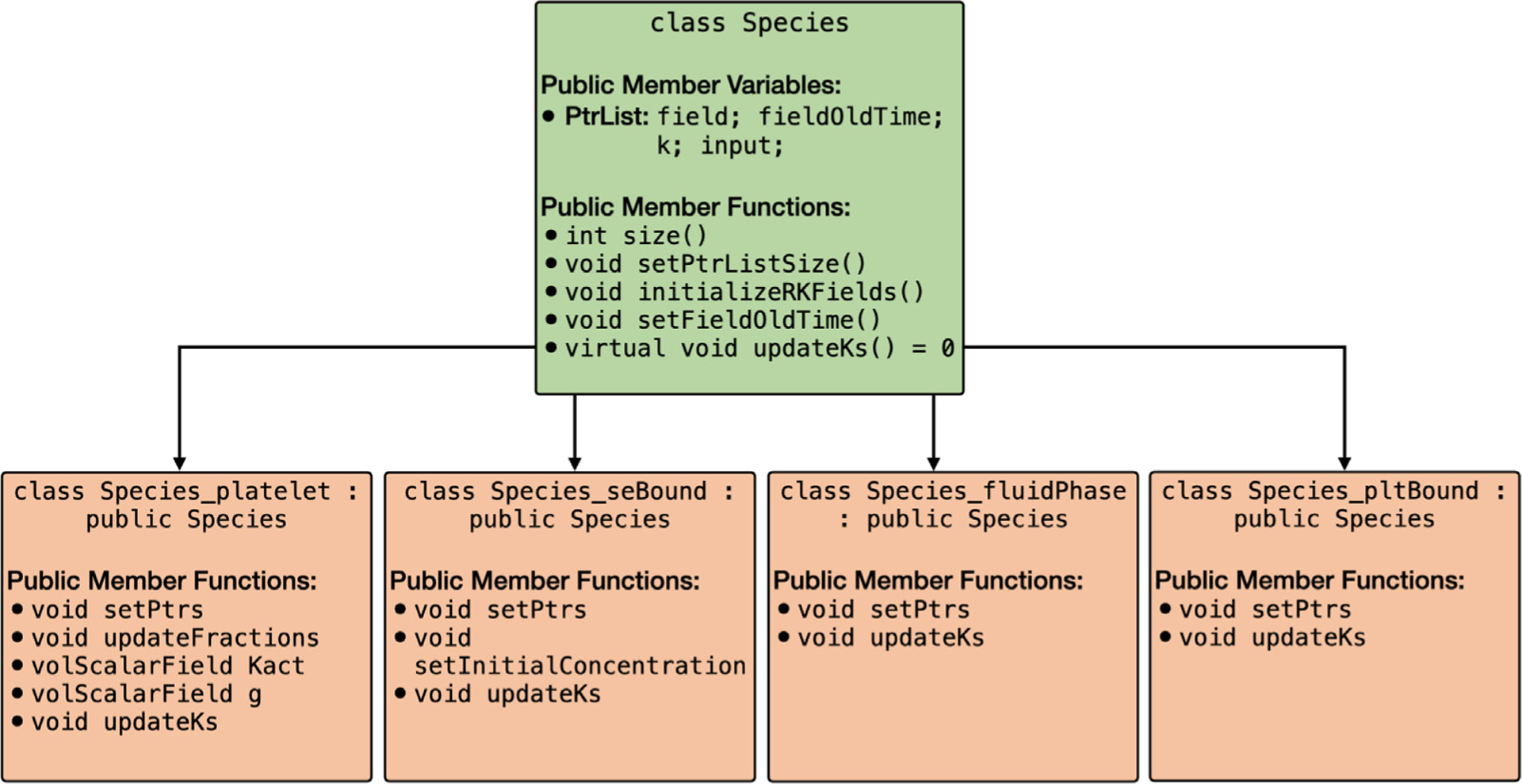
Graph of the Species class and derived classes Species_platelet, Species_seBound, Species_fluidPhase, Species_pltBound. All derived classes inherit the public member variables and public member functions defined in the Species class.

Models of blood clotting typically involve multiple platelet and biochemical species. For instance, the Leiderman-Fogelson model [3] consists of four platelet species and 50 biochemical species. To address this complexity, clotFoam has been developed to accommodate models with any number of platelet and biochemical species. These models are implemented following a polymorphic approach, with an abstract base class called Species from which four classes are derived to align with the species defined in the mathematical model: Species_platelet, Species_seBound, Species_fluidPhase, Species_plt Bound. The inheritance relationship of these classes is depicted in Fig. 4.

The Species object consists of four pointer lists to facilitate the management of the subspecies fields, including field values (solutions), previous field values (solutions from intermediate time steps used in the fractional-step method and RK4 solver), k values (computed for the RK4 solver), and the argument of the k values (inputs for the k values in the RK4 solver). The public member functions of the derived classes enable the setting of pointers to other Species objects and define the reaction functions specific to their corresponding subspecies. The function updateKs computes and retains the reaction term, $R_{i}$, for each subspecies, utilizing input parameters passed from the RK4 method. The initialization of every derived Species object occurs within the createFields.H file, and the pointers are set in setSpeciesPointers.H.

## 3.4. Adapting the framework for different clotting models

*clotFoam* was developed with the aim of promoting scientific research and reproducibility in the field of hemostasis, thrombosis, and clotting disorders. Thus, we illustrate the procedure for introducing a new species into the software. Consider the addition of a fluid-phase species I, that inhibits thrombin indefinitely. This reaction can be written as:

(11) $$I+E_{2}\overset{k_{I}}{\to }E_{2,\text{ inh }},$$

where $k_{I}$ is the association rate of inhibitor and enzyme, and $E_{2\text{,inh }}$ is the resulting inhibited thrombin. Although the addition of two new species, I and $E_{2,\text{ inh }}$, is necessary for incorporating the desired reaction into the software, we will focus our discussion on the steps involved in including just the inhibitor species as it applies to any additional species. The new reaction term that is added to the ADR equation for I is:

(12) $$\text{reaction rate}:-k_{I}IE_{2},$$

which fits the form of Eq. (6), with a reaction term defined by the right-hand side. The following sequential steps outline the procedure for adding I to the software:

1. Within the case directory $FOAM_CASE:
  - In the 0 directory:
    - Create a new field, such as fluidPhase_I.
  - In the constant/inputParameters file:
    - Update the number of fluid-phase species, num_fluidPhase.
    - Incorporate the parameter kI using appropriate units.
  - Within the *clotFoam* software:
  - In the chemConstants.H file:
    - Read in the parameter kI defined in the inputParameters dictionary.
  - In the Species_fluidPhase.H class file:
    - Update the reaction function updateKs to include
k[4]=-kI*I*E2
      where I is a reference to the input necessary for the RK4 solver,
      const volScalarField& I = input [4], defined at the beginning of the updateKs function.
    - If necessary, update the pointers in the constructor and in the setPtrs function (not required for this example).
  - In the createFields.H file:
    - Add the fluidPhase_I field to the 4th index of the PtrList called fluidPhase.field.
  - In the setSpeciesPointers.H file:
    - If required, update the fluidPhase.setPtrs argument corresponding to step 2(b)ii.

## 4. Illustrative examples

Here, we present two illustrative examples for which the code and instructions are included in the repository. These examples showcase the capabilities of the *clotFoam* solver in simulating blood clotting phenomena. The first example is a 2D thrombosis case based on previous results published by our group [3,16]. The second example is a 3D simulation of hemostasis that replicates the microfluidic device described in our previous study by Schoeman et al. [14]. Additional examples of convergence and validation can be found in the supplementary material.

### 4.1. Thrombosis in a rectangular channel

**Fig. 5.**
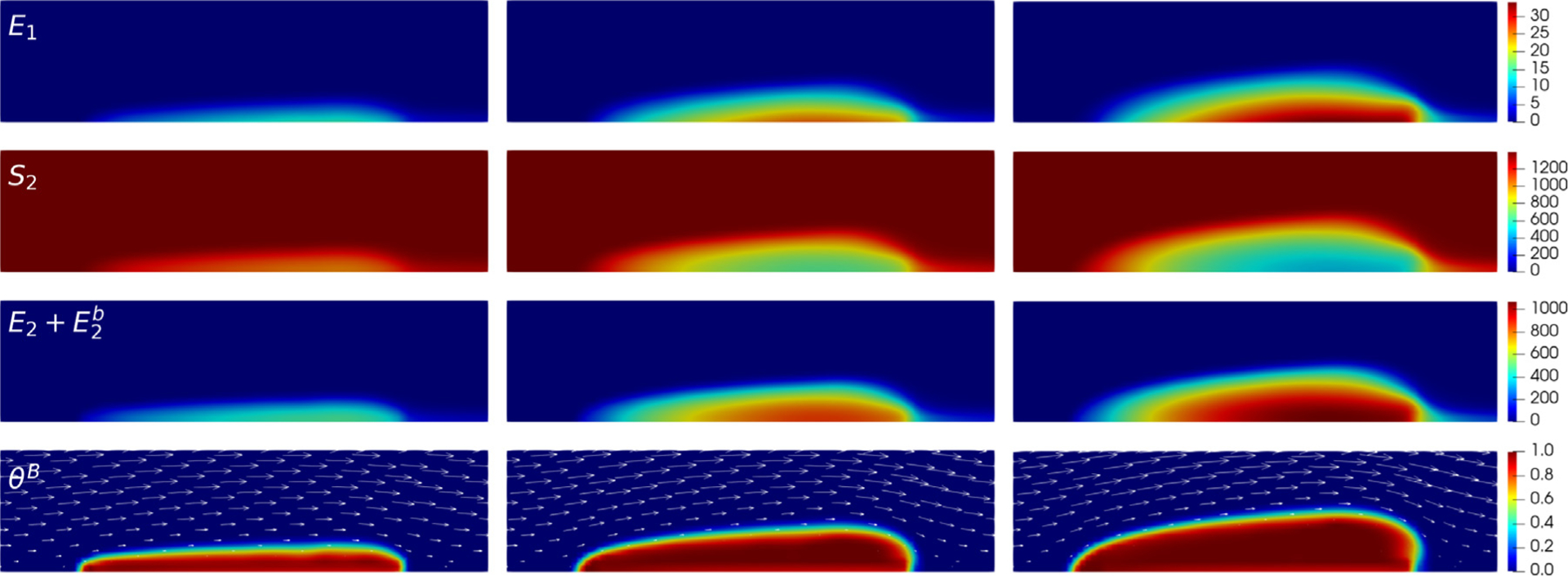
Close-up of 140 μm  long by 35 μm high region around the thrombus to view various species (rows) at times 200, 400 and 600 s in the left, middle and right columns, respectively. The first 3 rows show spatial concentrations (in nM) of E1 and S2 and the total thrombin concentration $\left(E_{2}+E_{2}^{b}\right)$ which was calculated in ParaView during post-processing. The bottom row shows the resulting thrombus growth via the bound platelet fraction in a dynamic fluid environment where the fluid velocity field is depicted by the white arrows.

To verify the reliability of *clotFoam* in modeling clotting phenomena, we compare its results with those in our previous work [3,16] and simulate clotting in a 240μm long by 60μm high rectangular channel with an approximately 90μm long adhesive and reactive patch centered on the bottom wall. The domain is discretized using a uniform mesh of (128 × 32) cells, which is divided into three blocks as depicted in Fig. 3. It should be noted that the coagulation reactions used in clotFoam are a simplified version of those used in our previous work, and therefore, the outcomes are not expected to be identical. Nevertheless, we demonstrate that clotFoam produces similar concentrations of bound thrombin, clot sizes and densities, all on the same timescale as in our previous studies [3,16]. This is illustrated in Fig. 5, where we show spatial distributions of substrates, enzymes, and clots formed after 200, 400, and 600 s of clotting activity.

### 4.2. Hemostasis in a microfluidic device

**Fig. 6.**
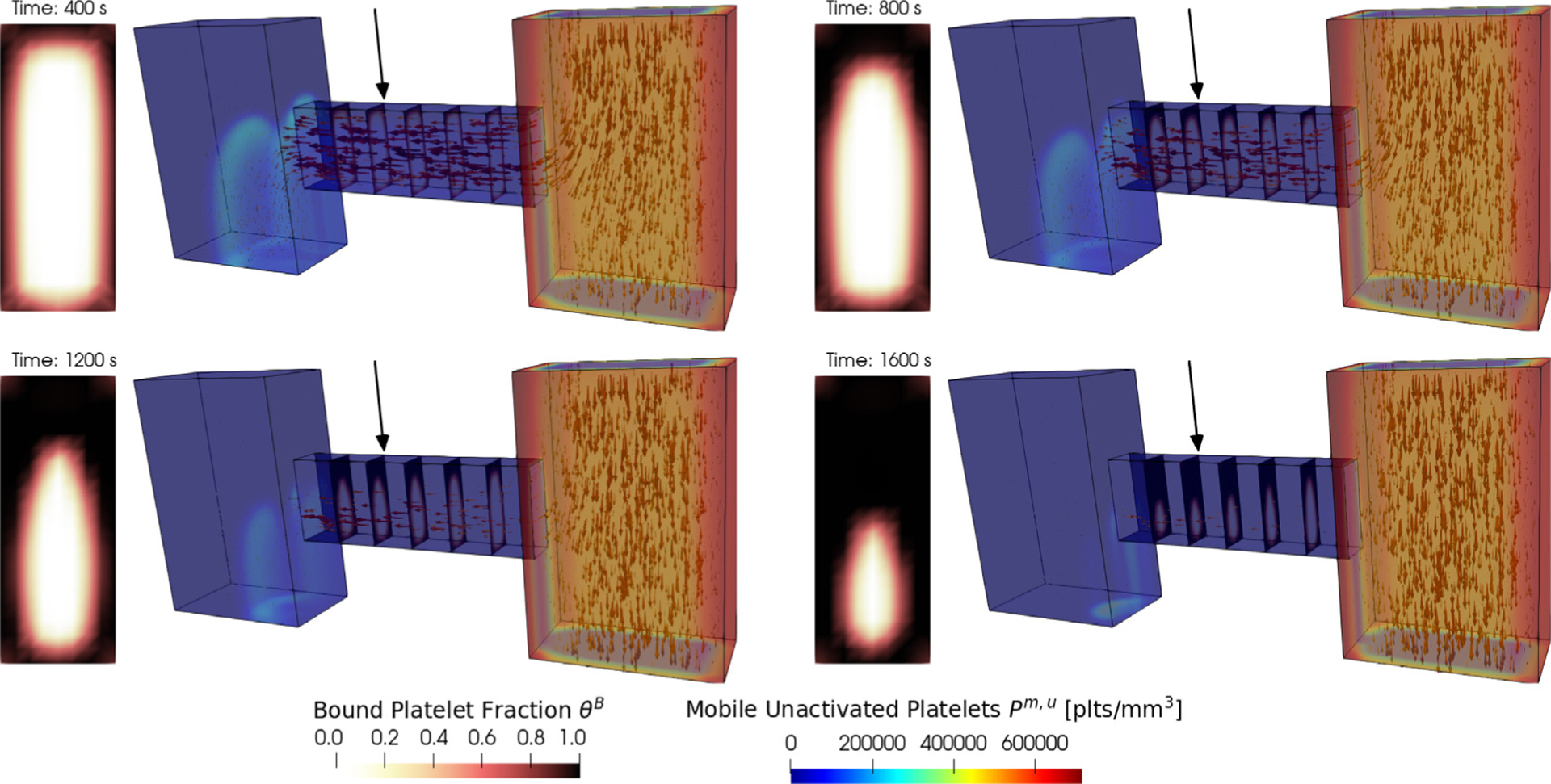
Snapshots of a *clotFoam* simulation of hemostasis in an H-shaped microfluidic device for time 400, 800, 1200, 1600 s. The simulation shows mobile platelets entering the right vertical channel and being transported by the fluid (indicated by arrows) through the horizontal injury channel. As they pass through the injury, they start to aggregate and form a platelet plug, which is visualized via the bound platelet fraction $\theta^{B}$ in the five slices within the injury channel.

To demonstrate the versatility of *clotFoam* in simulating clotting in various two and three dimensional domains, we have included a case that replicates the H-shaped microfluidic device used in Schoeman et al. [14] to model hemostasis. In their experiments, whole blood is introduced into the right “blood channel”, while a buffer fluid is introduced into the left vertical channel. The pressure difference between the channels causes blood to flow through the horizontal “injury channel”, which is coated with tissue factor and collagen proteins that initialize coagulation and platelet adhesion/aggregation respectively. Clots build up in the injury channel without restricting blood flow in the blood channel. Fig. 6 shows snapshots of the 3D domain at four different times during the clotting process. Due to difficulties visualizing the entire clot in the injury channel, we have displayed slices through the injury channel to highlight the spatial growth of the clot at various points down the injury channel. The rectangular plots to the left of each 3D domain are an enlarged view of the slice at the location indicated by the black arrow and show the clot distribution and its dynamics over time. The thrombus growth patterns observed in the experimental results presented by Schoeman et al. [14] show thrombus formation primarily at the front (right) of the injury channel while the thrombus generated by clotFoam, shown in Fig. 6, shows the buildup of thrombus closer to the back (left) of the injury channel. We hypothesize that this discrepancy may be attributed to the absence of shear dependence in the current platelet aggregation model, and this aspect will be investigated as part of our future work.

## 5. Impact and conclusions

Computational models that simulate clotting phenomena have provided a significant step toward a better understanding of hemostasis, thrombosis, and clotting disorders. In this work, we have presented *clotFoam*, an open-source software for simulating clotting using the computational fluid dynamics framework OpenFOAM. To demonstrate the reliability of *clotFoam*, we compared its outcomes with our previous computational work studying thrombosis in rectangular channels [3,14–16]. Our results show that *clotFoam* produces a similar clot structure and thrombin concentrations, thereby verifying its implementation of a reduced coagulation and platelet aggregation model. Furthermore, we demonstrated the versatility of *clotFoam* by simulating clotting in an H-shaped microfluidic device, which was used in experiments for modeling hemostasis. While the simulations did not replicate the experiments exactly, they illustrated the potential of *clotFoam* for investigating clotting in various microfluidic geometries.

In conclusion, *clotFoam* offers a reliable and flexible platform for simulating clotting phenomena. With the ability to manipulate the platelet and coagulation cascade models, researchers can use *clotFoam* to investigate various aspects of thrombus formation and design microfluidic devices for studying hemostasis. The open-source nature of *clotFoam* also allows for communitydriven development and improvement of the software, making it an accessible tool for researchers in the field.

## Supplementary Material

1

## Figures and Tables

**Fig. C.7.**
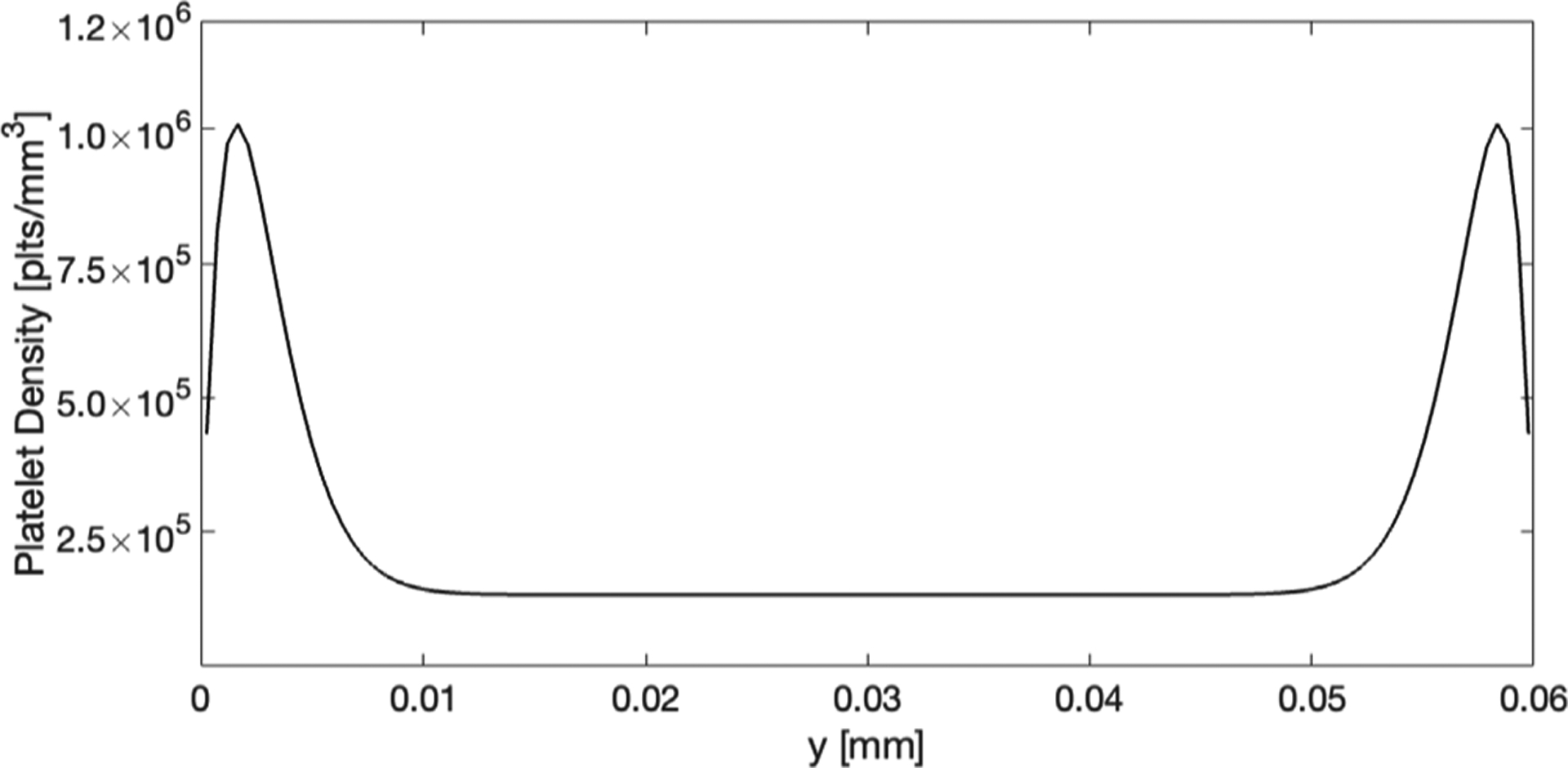
Inlet profile for the mobile-unactivated platelets in a vessel with r=30μm. The peak-to-center ratios is set to approximately 7.5 using an amplitude parameter of K=330.

## Data Availability

Data will be made available on request

## Appendix A. Platelet aggregation model

From our previous work, the number densities of the four platelet species are denoted by $P^{m,u},P^{m,a},P^{b,a},P^{b,se}$, where the superscripts represent mobile unactivated, mobile activated, platelet-bound activated, and subendothelium-bound activated respectively. Platelet aggregation is described by the following system of ADR equations:

(A.1)
$$\frac{\partial P^{m,u}}{\partial t}=-\underset{\;}{\underset{\text{Transport by advection and "diffusion"}}{\underset{︸}{\nabla \cdot \left\{W\left(\theta^{T}\right)\left(\vec{u}P^{m,u}-D_{P}\nabla P^{m,u}\right)\right\}}}}-\underset{\;}{\underset{\text{Adhesion to subendothelium}}{\underset{︸}{k_{\text{adh}}H_{\text{adh}}\left(\vec{x}\right)\left(P_{\text{max}}-P^{b,se}\right)P^{m,u}}}}-\underset{\;}{\underset{\text{Activation by ADP or thrombin}}{\underset{︸}{\left\{A_{1}\left(\left[\text{ADP}\right]\right)+A_{2}\left(E_{2}\right)\right\}P^{m,u}}}},$$

(A.2)
$$\frac{\partial P^{m,a}}{\partial t}=-\nabla \cdot \left\{W\left(\theta^{T}\right)\left(\vec{u}P^{m,a}-D_{P}\nabla P^{m,a}\right)\right\}-k_{\text{adh}}\left(\vec{x}\right)\left(P_{\text{max}}-P^{b,se}\right)P^{m,a}+\left\{A_{1}\left(\left[\text{ADP}\right]\right)+A_{2}\left(E_{2}\right)\right\}P^{m,u}-\underset{\;}{\underset{\text{Cohesion to bound platelets}}{\underset{︸}{k_{\text{coh}}g\left(\eta \right)P_{\text{max}}P^{m,a}}}},$$

(A.3)
$$\frac{\partial P^{b,a}}{\partial t}=-k_{adh}H_{adh}(\vec{x})\left(P_{max}-P^{b,se}\right)P^{b,a}+k_{coh}g(\eta )P_{max}P^{m,a},$$

(A.4)
$$\frac{\partial P^{b,se}}{\partial t}=k_{adh}H_{adh}(\vec{x})\left(P_{max}-P^{b,se}\right)\left(P^{m,u}+P^{m,a}+P^{b,a}\right).$$

The hindered transport function is a monotonically decreasing function defined as $W\left(\theta^{T}\right)=tanh\left(\pi \left(1-\theta^{T}\right)\right)$, where $\theta^{T}$ is the ratio of the sum of all platelet species to $P_{max}$. The function assumes that the transport of mobile platelets is only slightly hindered until $\theta^{T}$ approaches approximately 0.5, after which the transport of mobile platelets is drastically reduced. The adhesion function $H_{adh}(\vec{x})$ is chosen to be unity or zero, and defines the region where mobile platelets can stick to specified walls on the domain boundary.

Platelet-platelet cohesion is modeled through the parameters $k_{\text{coh }}$ and the binding-affinity function g(η), where the latter depends on a non-dimensional virtual substance η that is produced by bound platelets. In *clotFoam*, the virtual substance is modeled by diffusing the bound platelet fraction a distance of $L_{\eta }$ over an interval of Δt through the use of a discrete diffusion equation:

(A.5)
$$\frac{\eta -\eta_{0}}{\Delta t}=D_{\eta }\nabla^{2}\eta$$

where $\eta_{0}$ is the current known bound platelet fraction, $\eta_{0}=\theta_{n}^{B}$. The diffusion constant is defined as $D_{\eta }=\frac{L_{\eta }^{2}}{4\Delta t}$, and therefore (A.5) is implemented as:

(A.6)
$$\eta -\frac{L_{\eta }^{2}}{4}\nabla^{2}\eta =\theta_{n}^{B}$$

The definition of η presented above departs somewhat from the corresponding quantity in our previous work, and is intended to rectify the overly diffusive approach used in our previous work [3,15,16]. The binding affinity function is defined as:

(A.7)
$$g(\eta )=\frac{g_{0}{\left(\eta -\eta_{t}\right)}^{3}}{\eta_{*}^{3}+{\left(\eta -\eta_{t}\right)}^{3}}$$

where $\eta_{t}$ is a threshold value for which there is no binding, $\eta_{*}+\eta_{t}$ indicates the value of η for which g(η) changes rapidly, and $g_{0}=$ $\frac{\eta_{*}^{3}+{\left(1-\eta_{t}\right)}^{3}}{{\left(1-\eta_{t}\right)}^{3}}$ so that g(1)=1.

## Appendix B. Coagulation model

The reduced coagulation network is modeled by the following set of enzymatic reactions:

(B.1)
$$S_{1}+E_{0}\underset{k_{C_{0}}^{-}}{\overset{k_{C_{0}}^{+}}{⇌}}C_{0}\overset{k_{C_{0}}^{\text{cat }}}{⟶}E_{0}+E_{1}$$

(B.2)
$$S_{1}+P_{1}\underset{k_{S_{1}}^{-}}{\overset{k_{S_{1}}^{+}}{⇌}}S_{1}^{b}$$

(B.3)
$$E_{1}+P_{1}\underset{k_{E_{1}}^{-}}{\overset{k_{E_{1}}^{+}}{⇌}}E_{1}^{b}$$

(B.4)
$$S_{2}+P_{2}\underset{k_{S_{2}}^{-}}{\overset{k_{S_{2}}^{+}}{⇌}}S_{2}^{b}$$

(B.5)
$$E_{2}+P_{2}\underset{k_{E_{2}}^{-}}{\overset{k_{E_{2}}^{+}}{⇌}}E_{2}^{b}$$

(B.6)
$$S_{2}^{b}+E_{1}^{b}\underset{k_{C_{1}}^{-}}{\overset{k_{C_{1}}^{+}}{⇌}}C_{1}\overset{k_{C_{1}}^{\text{cat }}}{⟶}E_{1}^{b}+E_{2}^{b}$$

(B.7)
$$S_{1}^{b}+E_{2}^{b}\underset{k_{C_{2}}^{-}}{\overset{k_{C_{2}}^{+}}{⇌}}C_{2}\overset{k_{C_{2}}^{\text{cat }}}{⟶}E_{1}^{b}+E_{2}^{b}$$

Using the law of mass action, a system of twelve differential equations is derived to govern the reactions of the twelve biochemical species present in the coagulation cascade. These equations are categorized as fluid-phase, platelet-bound, and subendothelium-bound for organizational purposes. All concentrations of the biochemical species are measured in units of $nmol/{mm}^{3}$, and platelet densities are converted to concentrations using Avogadro’s constant $N_{A}=6.02214076\times 10^{14}{nmol}^{-1}$, where $N_{A}$ is the number of molecules in one mole of a substance.

### Fluid-phase:

(B.8)
$$\frac{\partial S_{1}}{\partial t}=\;\;-\nabla \cdot \left(\vec{u}S_{1}-D\nabla S_{1}\right)\;\;-k_{S_{1}}^{+}\left\{\frac{N_{1}}{N_{A}}\left(P^{b,a}+P^{se,a}\right)-\left(S_{1}^{b}+E_{1}^{b}+C_{1}+C_{2}\right)\right\}S_{1}+k_{S_{1}}^{-}S_{1}^{b},\;\text{ with }-{\left.D\overset{ˆ}{n}\cdot \nabla S_{1}\right|}_{\partial \Omega_{\text{inj }}}=-k_{C_{0}}^{+}S_{1}E_{0}+k_{C_{0}}^{-}C_{0},$$

(B.9)
$$\frac{\partial S_{2}}{\partial t}=-\nabla \cdot \left(\vec{u}S_{2}-D\nabla S_{2}\right)-k_{S_{2}}^{+}\left\{\frac{N_{2}}{N_{A}}\left(P^{b,a}+P^{se,a}\right)-\left(S_{2}^{b}+E_{2}^{b}+C_{1}+C_{2}\right)\right\}S_{2}+k_{S_{2}}^{-}S_{2}^{b},$$

(B.10)
$$\frac{\partial E_{1}}{\partial t}=-\nabla \cdot \left(\vec{u}E_{1}-D\nabla E_{1}\right)-k_{E_{1}}^{+}\left\{\frac{N_{1}}{N_{A}}\left(P^{b,a}+P^{se,a}\right)-\left(S_{1}^{b}+E_{1}^{b}+C_{1}+C_{2}\right)\right\}E_{1}+k_{E_{1}}^{-}E_{1}^{b},\text{with}-{\left.D\overset{ˆ}{n}\cdot \nabla E_{1}\right|}_{\partial \Omega_{inj}}=k_{C_{0}}^{\text{cat }}C_{0},$$

(B.11)
$$\frac{\partial E_{2}}{\partial t}=-\nabla \cdot \left(\vec{u}E_{2}-D\nabla E_{2}\right)\;-k_{E_{2}}^{+}\left\{\frac{N_{2}}{N_{A}}\left(P^{b,a}+P^{se,a}\right)-\left(S_{2}^{b}+E_{2}^{b}+C_{1}+C_{2}\right)\right\}E_{2}+k_{E_{2}}^{-}E_{2}^{b}.$$

### Platelet-bound:

(B.12)
$$\frac{\partial S_{1}^{b}}{\partial t}=\underset{\;}{\underset{\text{Binding and unbinding on platelet surface}}{\underset{︸}{k_{S_{1}}^{+}\left\{\frac{N_{1}}{N_{A}}\left(P^{b,a}+P^{se,a}\right)-\left(S_{1}^{b}+E_{1}^{b}+C_{1}+C_{2}\right)\right\}S_{1}-k_{S_{1}}^{-}S_{1}^{b}}}}-k_{C_{2}}^{+}S_{1}^{b}E_{2}^{b}+k_{C_{2}}^{-}C_{2},$$

(B.13)
$$\frac{\partial S_{2}^{b}}{\partial t}=k_{S_{2}}^{+}\{\underset{\begin{array}{c} \;\\ \; \end{array}}{\underset{\text{Total concentration of binding sites}}{\underset{︸}{\frac{N_{2}}{N_{A}}\left(P^{b,a}+P^{Se,a}\right)}}}-\underset{\begin{array}{c} \;\\ \; \end{array}}{\underset{\text{Occupied binding sites }}{\underset{︸}{\left(S_{2}^{b}+E_{2}^{b}+C_{1}+C_{2}\right)}}}\}S_{2}-k_{S_{2}}^{-}S_{2}^{b}-k_{C_{1}}^{+}S_{2}^{b}E_{1}^{b}+k_{C_{1}}^{-}C_{1},$$

(B.14)
$$\frac{\partial E_{1}^{b}}{\partial t}=k_{E_{1}}^{+}\left\{\frac{N_{1}}{N_{A}}\left(P^{b,a}+P^{se,a}\right)-\left(S_{1}^{b}+E_{1}^{b}+C_{1}+C_{2}\right)\right\}E_{1}-k_{E_{1}}^{-}E_{1}^{b}-k_{C_{1}}^{+}S_{2}^{b}E_{1}^{b}+\left(k_{C_{1}}^{-}+k_{C_{1}}^{\text{cat }}\right)C_{1}+\underset{\begin{array}{c} \;\\ \; \end{array}}{\underset{\text{Positive feedback}}{\underset{︸}{k_{C_{2}}^{\text{cat }}C_{2}}}},$$

(B.15)
$$\frac{\partial E_{2}^{b}}{\partial t}=k_{E_{2}}^{+}\left\{\frac{N_{2}}{N_{A}}\left(P^{b,a}+P^{se,a}\right)-\left(S_{2}^{b}+E_{2}^{b}+C_{1}+C_{2}\right)\right\}E_{2}-k_{E_{2}}^{-}E_{2}^{b}-k_{C_{2}}^{+}S_{1}^{b}E_{2}^{b}+\left(k_{C_{2}}^{-}+k_{C_{2}}^{\text{cat }}\right)C_{2}+\underset{\begin{array}{c} \;\\ \; \end{array}}{\underset{\text{Positive feedback}}{\underset{︸}{k_{C_{1}}^{\text{cat }}C_{1}}}},$$

(B.16)
$$\frac{\partial C_{1}}{\partial t}=k_{C_{1}}^{+}S_{2}^{b}E_{1}^{b}-\left(k_{C_{1}}^{-}+k_{C_{1}}^{\text{cat }}\right)C_{1},$$

(B.17)
$$\frac{\partial C_{2}}{\partial t}=k_{C_{2}}^{+}S_{1}^{b}E_{2}^{b}-\left(k_{C_{2}}^{-}+k_{C_{2}}^{\text{cat }}\right)C_{2}.$$

### Subendothelium-bound:

(B.18)
$$\frac{\partial E_{0}}{\partial t}=-k_{C_{0}}^{+}S_{1}E_{0}+\left(k_{C_{0}}^{-}+k_{C_{0}}^{\text{cat }}\right)C_{0},\;\;on\;\partial \Omega_{inj},$$

(B.19)
$$\frac{\partial C_{0}}{\partial t}=k_{C_{0}}^{+}S_{1}E_{0}-\left(k_{C_{0}}^{-}+k_{C_{0}}^{\text{cat }}\right)C_{0},\;\;on\;\partial \Omega_{inj}.$$

## Appendix C. Boundary conditions

The boundary conditions for most variables remain consistent across different geometries. The variables that experience changes in their boundary conditions when the geometry is altered are those introduced at the domain inlet, such as the fluid velocity $\vec{u}$, the mobile unactivated platelets $P^{m,u}$, and the kinematic pressure, $\tilde{p}=p/\rho$, at the outlet. The specific conditions for these variables are detailed in the corresponding subsections specific to each domain. For all other variables, the boundary conditions are applied according to Table C.1.

### C.1. Boundary conditions for thrombosis in a rectangular channel

The velocity profile for $\vec{u}={\left[\begin{array}{ll} u & v \end{array}\right]}^{T}$ is prescribed using the following function, with a shear rate of $\dot{\gamma }=1000{\;s}^{-1}$ and radius =30μm:

(C.1)
$$u(y)=-\frac{\dot{\gamma }}{2r}(y-r{)}^{2}+\frac{1}{2}\dot{\gamma }r.$$

This parabolic profile is implemented in OpenFOAM using the codedFixedValue boundary condition

**Table C.1:** Boundary conditions for each variable in the simulation. The table specifies the mathematical boundary condition and the corresponding implementation in OpenFOAM for each boundary. Some variables with the same boundary conditions have been grouped together for brevity.

Variable(s)	inlet	outlet	fixedWalls	injuryWalls
$\vec{u}$	*see below*	$\begin{array}{l} \frac{\partial \vec{u}}{\partial \overset{ˆ}{n}}=0\\ \; \end{array}$ zeroGradient	$\begin{array}{l} \vec{u}=0\\ \; \end{array}$ noSlip	$\begin{array}{l} \vec{u}=0\\ \; \end{array}$ noSlip
$\tilde{p}$	$\begin{array}{l} \frac{\partial \tilde{p}}{\partial \overset{ˆ}{n}}=0\\ \; \end{array}$ zeroGradient	$\begin{array}{l} \tilde{p}=p_{\text{out }} \end{array}$ fixedValue	$\begin{array}{l} \frac{\partial \tilde{p}}{\partial \overset{ˆ}{n}}=0\\ \; \end{array}$ zeroGradient	$\begin{array}{l} \frac{\partial \tilde{p}}{\partial \overset{ˆ}{n}}=0 \end{array}$ zeroGradient
$P^{m,u}$	*see below* codedFixedValue	$\begin{array}{l} \frac{\partial P^{m,u}}{\partial \overset{ˆ}{n}}=0 \end{array}$ zeroGradient	$\begin{array}{l} \frac{\partial P^{m,u}}{\partial \overset{ˆ}{n}}=0\\ \; \end{array}$ zeroGradient	$\begin{array}{l} \frac{\partial P^{m,u}}{\partial \overset{ˆ}{n}}=0\\ \; \end{array}$ zeroGradient
$\begin{array}{l} P^{m,a}\\ P^{b,a}\\ P^{b,se} \end{array}$	$\begin{array}{l} \frac{\partial P^{k}}{\partial \overset{ˆ}{n}}=0 \end{array}$ zeroGradient	$\begin{array}{l} \frac{\partial P^{k}}{\partial \overset{ˆ}{n}}=0 \end{array}$ zeroGradient	$\begin{array}{l} \frac{\partial P^{k}}{\partial \overset{ˆ}{n}}=0 \end{array}$ zeroGradient	$\begin{array}{l} \frac{\partial P^{k}}{\partial \overset{ˆ}{n}}=0\\ \; \end{array}$ zeroGradient
$S_{1}$	$\begin{array}{l} S_{1}=\left(\begin{array}{c} \text{ normal }\\ \text{ concentration } \end{array}\right) \end{array}$ fixedValue	$\begin{array}{l} \frac{\partial S_{1}}{\partial \overset{ˆ}{n}}=0\\ \; \end{array}$ zeroGradient	$\begin{array}{l} \frac{\partial S_{1}}{\partial \overset{ˆ}{n}}=0 \end{array}$ zeroGradient	$\begin{array}{l} -D\frac{\partial S_{1}}{\partial \overset{ˆ}{n}}=-k_{C_{0}}^{+}S_{1}+k_{C_{0}}^{-}C_{0} \end{array}$ codedMixed
$E_{1}$	$\begin{array}{l} \frac{\partial E_{1}}{\partial \overset{ˆ}{n}}=0 \end{array}$ zeroGradient	$\begin{array}{l} \frac{\partial E_{1}}{\partial \overset{ˆ}{n}}=0\\ \; \end{array}$ zeroGradient	$\begin{array}{l} \frac{\partial E_{1}}{\partial \overset{ˆ}{n}}=0\\ \; \end{array}$ zeroGradient	$\begin{array}{l} -D\frac{\partial E_{1}}{\partial \overset{ˆ}{n}}=k_{C_{0}}^{\text{cat }}C_{0}\\ \; \end{array}$ codedMixed
$S_{2}$	$\begin{array}{l} S_{2}=\left(\begin{array}{c} \text{ normal }\\ \text{ concentration } \end{array}\right) \end{array}$ fixedValue	$\begin{array}{l} \frac{\partial S_{2}}{\partial \overset{ˆ}{n}}=0 \end{array}$ zeroGradient	$\begin{array}{l} \frac{\partial S_{2}}{\partial \overset{ˆ}{n}}=0 \end{array}$ zeroGradient	$\begin{array}{l} \frac{\partial S_{2}}{\partial \overset{ˆ}{n}}=0 \end{array}$ zeroGradient
$E_{2}$	$\begin{array}{l} \frac{\partial E_{2}}{\partial \overset{ˆ}{n}}=0 \end{array}$ zeroGradient	$\begin{array}{l} \frac{\partial E_{2}}{\partial \overset{ˆ}{n}}=0 \end{array}$ zeroGradient	$\begin{array}{l} \frac{\partial E_{2}}{\partial \overset{ˆ}{n}}=0\\ \; \end{array}$ zeroGradient	$\begin{array}{l} \frac{\partial E_{2}}{\partial \overset{ˆ}{n}}=0\\ \; \end{array}$ zeroGradient
$\begin{array}{l} S_{1}^{b}\\ S_{2}^{b}\\ E_{1}^{b}\\ E_{2}^{b}\\ C_{1}\\ C_{2} \end{array}$	$\begin{array}{l} \frac{\partial C^{k}}{\partial \overset{ˆ}{n}}=0\\ \; \end{array}$ zeroGradient	$\begin{array}{l} \frac{\partial C^{k}}{\partial \overset{ˆ}{n}}=0\\ \; \end{array}$ zeroGradient	$\begin{array}{l} \frac{\partial C^{k}}{\partial \overset{ˆ}{n}}=0\\ \; \end{array}$ zeroGradient	$\begin{array}{l} \frac{\partial C^{k}}{\partial \overset{ˆ}{n}}=0 \end{array}$ zeroGradient
$\begin{array}{l} E_{0}\\ C_{0} \end{array}$	$\begin{array}{l} \frac{\partial C^{k}}{\partial \overset{ˆ}{n}}=0\\ \; \end{array}$ zeroGradient	$\begin{array}{l} \frac{\partial C^{k}}{\partial \overset{ˆ}{n}}=0\\ \; \end{array}$ zeroGradient	$\begin{array}{l} \frac{\partial C^{k}}{\partial \overset{ˆ}{n}}=0\\ \; \end{array}$ zeroGradient	See equations (B.18) and (B.19) solved in odeSolver.H
ADP	$\begin{array}{l} \frac{\partial \left[ADP\right]}{\partial \overset{ˆ}{n}}=0 \end{array}$ zeroGradient	$\begin{array}{l} \frac{\partial \left[ADP\right]}{\partial \overset{ˆ}{n}}=0\\ \; \end{array}$ zeroGradient	$\begin{array}{l} \frac{\partial \left[ADP\right]}{\partial \overset{ˆ}{n}}=0 \end{array}$ zeroGradient	$\begin{array}{l} \frac{\partial [ADP]}{\partial \overset{ˆ}{n}}=0 \end{array}$ zeroGradient

The mobile-unactivated platelets $P^{m,u}$ exhibit margination behavior at the inlet, where the concentration near the wall is higher compared to the center of the vessel. To capture this phenomenon, we employ a shape function based on the inlet profile proposed by Eckstein and Belgacem [24]. The inlet profile for mobile-unactivated platelets entering a 2D computational domain $\Omega =\left[x_{0},x_{max}\right]\times \left[y_{0},y_{max}\right]$ is described by:

(C.2)
$$P^{m,u}\left(x_{0},y,t\right)=P_{0}c(y),$$

where $P_{0}$ is the normal density of platelets. The shape function c(y) is defined as:

(C.3)
$$c(y)=C_{0}\left[1+KR(y,r{)}^{m-1}(1-R(y,r){)}^{n-1}\right].$$

with $C_{0}$ being a normalizing parameter, K determining the relative amplitude of the shape, and m,n∈N. The function $R\left(y,r\right)$ is given by:

(C.4)
$$R(y,r)=\frac{\left|y-r\right|}{r},$$

where the vessel radius is $r=\left(y_{max}-y_{0}\right)/2$. The exponents are set as m=19 and n=2 to produce the effect of a near-wall peak added to a uniform density $P_{0}$. The normalization parameter is defined as:

(C.5)
$$\frac{1}{C_{0}}=\frac{1}{2r}\int_{y_{0}}^{y_{max}}\left[1+KR(y,r{)}^{m-1}(1-R(y,r){)}^{n-1}\right]dy$$

Fig. C.7 shows the specific inlet profile used in the thrombosis example in Section 4.1, where r=30μm and K=330.

**Table C.2:** Fluid velocity and kinematic pressure boundary conditions.

Left inlet $\left(Q_{\text{left }}\right)$	$1.17\times 10^{-2}{\;mm}^{3}/s$
Right inlet $\left(Q_{\text{right }}\right)$	$9.32\times 10^{-2}{\;mm}^{3}/s$
Left outlet $\left({\tilde{p}}_{\text{left }}\right)$	$1.11\times 10^{6}{\;mm}^{2}/s^{2}$
Right outlet $\left({\tilde{p}}_{\text{right }}\right)$	$1.78\times 10^{6}{\;mm}^{2}/s^{2}$

### C.2. Boundary conditions for hemostasis in a microfluidic device

The velocity profile $\vec{u}={\left[\begin{array}{lll} u & v & w \end{array}\right]}^{T}$ is prescribed using the flowRateInletVelocity boundary condition, with volumetric flow rates $Q_{\text{left }}$ and $Q_{\text{right }}$ at the upper left and upper right inlets, respectively. The kinematic pressure at the lower outlets is denoted by ${\tilde{p}}_{\text{left }}$ and ${\tilde{p}}_{\text{right. }}$. The specific values for the flow rates and kinematic pressure, adapted from Schoeman et al. [14], are provided in Table C.2.

The shape function Eq. (C.2) for $P^{m,u}$ is adapted for a 3D channel, $\Omega =\left[x_{0},x_{max}\right]\times \left[y_{0},y_{max}\right]\times \left[z_{0},z_{max}\right]$, and the normalization constant $C_{0}$ is calculated in two dimensions. Specifically, the shape function c(x,z) is defined by:

$$c\left(x,z\right)=C_{0}[1+K_{x}R{\left(x,r_{x}\right)}^{m-1}{\left(1-R\left(x,r_{x}\right)\right)}^{n-1}+K_{z}R{\left(z,r_{z}\right)}^{m-1}{\left(1-R\left(z,r_{z}\right)\right)}^{n-1}]$$

In the 3D hemostasis example, the amplitude parameters are set to $K_{x}=K_{z}=547$ to create a peak-to-center ratio of approximately 11.9. The exponents are set to m=19 and n=2.

## Appendix D. Discretization schemes

The discretization schemes employed in this work are summarized in Table D.3. To limit spurious oscillations caused by steep gradients at the thrombus edges, the van Leer flux limiter [25] is applied in the advective terms for platelets and biochemical species. In Section 3.2, we provided a detailed discussion on the choice of interpolation scheme for the hindered transport function $W\left(\theta^{T}\right)$, which plays a crucial role in the simulation. In the table below, Theta_Tfa and Theta_Tfd represent the face values of $\theta^{T}$ used for hindered advection and hindered diffusion, respectively. Both simulations employ a variable time step Δt that is set to ensure the Courant number, maxCo, does not exceed 0.75. For more practical examples and further information, please refer to the tutorials directory within the *clotFoam* repository on GitHub.

**Table D.3:** Discretization schemes for orthogonal meshes in *clotFoam*.

ddtSchemes	default	CrankNicolson 0.9;
	ddt(phi)	CrankNicolson 0.9;
gradSchemes	default	Gauss linear;
	grad(p)	Gauss linear;
divSchemes	default	none;
	div(phi,U)	Gauss linear;
	div(phiPlt,Plt)	Gauss limitedVanLeer 0.0 $Pmax;
	div(phi,chems)	Gauss vanLeer;
laplacianSchemes	default	Gauss linear orthogonal;
interpolationSchemes	default	linear
	interpolate(Theta_Tfa)	downwind phi;
	interpolate(Theta_Tfd)	localMax;
snGradSchemes	default	orthogonal;

## Appendix E. Parameter values

All of the parameters are from various sources as described in Leiderman & Fogelson [3] (see Tables E.4–E.9).

**Table E.4:** Fluid equation parameters.

Fluid density (ρ)	$1.0\times 10^{-3}\;g/{mm}^{3}$
Dynamic viscosity (μ)	$2.62507\times 10^{-3}\;g/mm/s$
Kinematic viscosity (v)	$2.62507{\;mm}^{2}/s$
Carman-Kozeny constant $\left(C_{CK}\right)$	$1.0\times 10^{6}{\;mm}^{-2}$

**Table E.5:** Diffusion coefficients by species.

Platelets $\left(D_{P}\right)$	$2.5\times 10^{-5}{\;mm}^{2}/s$
ADP $\left(D_{\text{ADP }}\right)$	$5\times 10^{-4}{\;mm}^{2}/s$
All other chemical species (D)	$5\times 10^{-5}{\;mm}^{2}/s$

**Table E.6:** Platelet equation parameters.

Platelet diameter $\left(P_{\text{diam }}\right)$	$3.0\times 10^{-3}\;mm$
Maximum packing density $\left(P_{max}\right)$	$6.67\times 10^{7}\;platelets/{mm}^{3}$
Normal density of platelets $\left(P_{0}\right)$	$2.5\times 10^{5}\;platelets/{mm}^{3}$
Adhesion rate $\left(k_{\text{adh }}\right)$	$3.3212\times 10^{-8}{\;mm}^{3}/s$
Cohesion rate $\left(k_{\text{coh }}\times P_{\text{max }}\right)$	$1.0\times 10^{4}{\;s}^{-1}$
Threshold for binding affinity $\left(\eta_{t}\right)$	$1.0\times 10^{-1}$
Rapid change in binding affinity $\left(\eta^{*}+\eta_{t}\right)$	$0.5-\eta_{t}$
Length of diffusion for the virtual substance $\left(L_{\eta }\right)$	$2.0\times P_{\text{diam }}$
Rate of activation by ADP $\left(k_{\text{adp }}^{\text{pla }}\right)$	$0.34{\;s}^{-1}$
Rate of activation by thrombin $\left(k_{e_{2}}^{\text{pla }}\right)$	$0.5{\;s}^{-1}$
Critical concentration of ADP $\left([\text{ ADP }{]}^{*}\right)$	$2.0\times 10^{-3}nmol\;$ of ADP per mm^3^
Critical concentration of thrombin $\left(e_{2}^{*}\right)$	$1.0\times 10^{-6}nmol\;$ of $e_{2}$ per mm^3^
Total ADP released $\left(\overset{ˆ}{A}\right)$	$2.0\times 10^{-8}nmol$ of ADP per platelet
Total time of ADP secretion $\left(\tau_{F}\right)$	6 s
Step size for calculating ADP secretion (Δτ)	0.25 s

**Table E.7:** Normal concentrations and surface binding site numbers.

Chemical	Concentration (clotFOAM)	Concentration	Binding Sites
$E_{0}$ (TF:VIIa)	$1.5\times 10^{-7}nmol/{mm}^{2}$	$15fmol/{cm}^{2}$	
$E_{1}$ (Xa)			$N_{1}=2700$
$E_{2}$ (Thrombin)			$N_{2}=2000$
$S_{1}\;(X)$	$1.7\times 10^{-4}nmol/{mm}^{3}$	0.17μM	$N_{1}=2700$
$S_{2}$ (II)	$1.4\times 10^{-3}nmol/{mm}^{3}$	1.4μM	$N_{2}=2000$

**Table E.8:** Reactions on subendothelium and platelet surface.

Reaction	Complex	Product	$\begin{array}{l} {mm}^{3}{nmol}^{-1}{\;s}^{-1} \end{array}$	$M^{-1}{\;s}^{-1}$	${\;s}^{-1}$	${\;s}^{-1}$
Activation (of-, by-)			(clotFOAM)			
$\left(S_{1},E_{0}\right)$	$C_{0}$	$E_{1}$	$k_{C_{0}}^{+}=8.95\times 10^{3}$	$k_{C_{0}}^{+}=8.95\times 10^{6}$	$k_{C_{0}}^{-}=1.0$	$k_{C_{0}}^{\text{cat }}=1.15$
$\left(S_{2}^{b},E_{1}^{b}\right)$	$C_{1}$	$E_{2}^{b}$	$k_{C_{1}}^{+}=1.03\times 10^{5}$	$k_{C_{1}}^{+}=1.03\times 10^{8}$	$k_{C_{1}}^{-}=1.0$	$k_{C_{1}}^{\text{cat }}=30.0$
$\left(S_{1}^{b},E_{2}^{b}\right)$	$C_{2}$	$E_{1}^{b}$	$k_{C_{2}}^{+}=1.73\times 10^{4}$	$k_{C_{2}}^{+}=1.73\times 10^{7}$	$k_{C_{2}}^{-}=1.0$	$k_{C_{2}}^{\text{cat }}=0.23$

**Table E.9:** Binding on platelet surface.

Reaction	Reactants	Product	$\begin{array}{l} {mm}^{3}{nmol}^{-1}{\;s}^{-1} \end{array}$ (clotFOAM)	$M^{-1}{\;s}^{-1}$	${\;s}^{-1}$
$S_{1}(\;V)$	$S_{1},P_{1}$	$S_{1}^{b}$	$k_{S_{1}}^{+}=5.7\times 10^{4}$	$k_{S_{1}}^{+}=5.7\times 10^{7}$	$k_{S_{1}}^{-}=0.17$
$E_{1}(Xa)$	$E_{1},P_{1}$	$E_{1}^{b}$	$k_{E_{1}}^{+}=1.0\times 10^{4}$	$k_{E_{1}}^{+}=1.0\times 10^{7}$	$k_{E_{1}}^{-}=2.5\times 10^{-2}$
$S_{2\;}(II)$	$S_{2},P_{2}$	$S_{2}^{b}$	$k_{S_{2}}^{+}=1.0\times 10^{4}$	$k_{S_{2}}^{+}=1.0\times 10^{7}$	$k_{S_{2}}^{-}=5.9$
$E_{2}\;(IIa)$	$E_{2},P_{2}$	$E_{2}^{b}$	$k_{E_{2}}^{+}=1.0\times 10^{4}$	$k_{E_{2}}^{+}=1.0\times 10^{7}$	$k_{E_{2}}^{-}=5.9$

## References

[1] Fogelson AL, Kuharsky AL. Membrane binding-site density can modulate activation thresholds in enzyme systems. J Theoret Biol 1998;193(1):1–18. 10.1006/jtbi.1998.0670. 9689939

[2] Kuharsky AL, Fogelson AL. Surface-mediated control of blood coagulation: The role of binding site densities and platelet deposition. Biophys J 2001;80(3):1050–74. 10.1016/S0006-3495(01)76085-7. 11222273 PMC1301304

[3] Leiderman K, Fogelson AL. Grow with the flow: A spatial-temporal model of platelet deposition and blood coagulation under flow. Math Med Biol: J IMA 2011;28(1):47–84. 10.1093/imammb/dqq005. PMC3499081 20439306

[4] Miyazawa K, Fogelson AL, Leiderman K. Inhibition of platelet-surfacebound proteins during coagulation under flow I: Antithrombin and heparin. Biophys J 2023;122(1):230–40. 10.1016/j.bpj.2022.11.023. 36325617 PMC9822793

[5] Neeves KB, Leiderman K. Mathematical models of hemostasis. In: Trauma induced coagulopathy. Springer; 2016, p. 567–84. 10.1007/978-3-319-28308-1_35.

[6] Leiderman K, Bannish B, Kelley M, Palmisano A. Mathematical models of thrombus formation and fibrinolysis. In: Cardiovascular thrombus: from pathology and clinical presentation to imaging, pharmacotherapy and interventions. Academic Press San Diego; 2018, p. 67–82. 10.1016/B978-0-12-812615-8.00005-3.

[7] Diamond SL. Systems biology of coagulation. J Thrombosis Haemostasis 2013;11:224–32. 10.1111/jth.12220. PMC3713523 23809126

[8] Yesudasan S, Averett RD. Recent advances in computational modeling of fibrin clot formation: A review. Comput Biol Chem 2019;83:107148. 10.1016/j.compbiolchem.2019.107148. 31751883 PMC6918949

[9] Anand M, Panteleev M, Ataullakhanov F. Computational models of hemostasis: Degrees of complexity. Appl Eng Sci 2022;10:100103. 10.1016/j.apples.2022.100103.

[10] Taylor JO, Meyer RS, Deutsch S, Manning KB. Development of a computational model for macroscopic predictions of device-induced thrombosis. Biomech Model Mechanobiol 2016;15:1713–31. 10.1007/s10237-016-0793-2. 27169403

[11] Govindarajan V, Zhu S, Li R, Lu Y, Diamond SL, Reifman J, Impact of tissue factor localization on blood clot structure and resistance under venous shear. Biophys J 2018;114(4):978–91. 10.1016/j.bpj.2017.12.034. 29490257 PMC5984989

[12] Méndez Rojano R, Zhussupbekov M, Antaki JF, Lucor D. Uncertainty quantification of a thrombosis model considering the clotting assay PFA-100^®^. Int J Numer Methods Biomed Eng 2022;38(5):e3595. 10.1002/cnm.3595. 35338596

[13] Bouchnita A, Belyaev AV, Volpert V. Multiphase continuum modeling of thrombosis in aneurysms and recirculation zones. Phys Fluids 2021;33(9):093314. 10.1063/5.0057393.

[14] Schoeman RM, Rana K, Danes N, Lehmann M, Di Paola JA, Fogelson AL, A microfluidic model of hemostasis sensitive to platelet function and coagulation. Cell Mol Bioeng 2017;10:3–15. 10.1007/s12195-016-0469-0. 28529666 PMC5435378

[15] Danes NA, Leiderman K. A density-dependent FEM-FCT algorithm with application to modeling platelet aggregation. Int J Numer Methods Biomed Eng 2019;35(9):e3212. 10.1002/cnm.3212. PMC6718345 31117155

[16] Leiderman K, Fogelson AL. The influence of hindered transport on the development of platelet thrombi under flow. Bull Math Biol 2013;75:1255–83. 10.1007/s11538-012-9784-3. 23097125 PMC6097848

[17] Rezaeimoghaddam M, van de Vosse FN. Continuum modeling of thrombus formation and growth under different shear rates. J Biomech 2022;132:110915. 10.1016/j.jbiomech.2021.110915. 35032838

[18] Wu W-T, Jamiolkowski MA, Wagner WR, Aubry N, Massoudi M, Antaki JF. Multi-constituent simulation of thrombus deposition. Sci Rep 2017;7(1):1–16. 10.1038/srep42720. 28218279 PMC5316946

[19] Greenshields C OpenFOAM V9 User Guide. London, UK: The OpenFOAM Foundation; 2021, URL https://doc.cfd.direct/openfoam/user-guide-v9.

[20] Shankar KN, Zhang Y, Sinno T, Diamond SL. A three-dimensional multiscale model for the prediction of thrombus growth under flow with singleplatelet resolution. PLoS Comput Biol 2022;18(1):e1009850. 10.1371/journal.pcbi.1009850. 35089923 PMC8827456

[21] Méndez Rojano R, Lai A, Zhussupbekov M, Burgreen GW, Cook K, Antaki JF. A fibrin enhanced thrombosis model for medical devices operating at low shear regimes or large surface areas. PLoS Comput Biol 2022;18(10):e1010277. 10.1371/journal.pcbi.1010277. 36190991 PMC9560616

[22] Issa RI. Solution of the implicitly discretised fluid flow equations by operator-splitting. J Comput Phys 1986;62(1):40–65. 10.1016/0021-9991(86)90099-9.

[23] Félétou M The endothelium, Part I: Multiple functions of the endothelial cells-focus on endothelium-derived vasoactive mediators. Biota Publishing; 2011, URL https://www.ncbi.nlm.nih.gov/books/NBK57145/. 21850763

[24] Eckstein EC, Belgacem F. Model of platelet transport in flowing blood with drift and diffusion terms. Biophys J 1991;60(1):53–69. 10.1016/S0006-3495(91)82030-6. 1883945 PMC1260038

[25] van Leer B Towards the ultimate conservative difference scheme. II. Monotonicity and conservation combined in a second-order scheme. J Comput Phys 1974;14(4):361–70. 10.1016/0021-9991(74)90019-9.

